# TSP-1 interaction with RANK and OPG: implications for bone remodeling and osteolytic bone metastasis

**DOI:** 10.1038/s41419-026-08600-9

**Published:** 2026-03-21

**Authors:** Laura Carminati, Fabio Sangalli, Chiara Urbinati, Elisa Longhi, Giulia Tomasoni, Patrizia Borsotti, Kim S. Midwood, Elena Carlessi, Marco Rusnati, Giulia Taraboletti

**Affiliations:** 1 Laboratory of Tumor Microenvironment, Department of Experimental Oncology, Istituto di Ricerche Farmacologiche Mario Negri IRCCS, Bergamo, Italy; 2 Laboratory of Medical Imaging, Department of Biomedical Engineering, Istituto di Ricerche Farmacologiche Mario Negri IRCCS, Bergamo, Italy; 3 Department of Molecular and Translational Medicine, University of Brescia, Brescia, Italy; 4 Kennedy Institute of Rheumatology, University of Oxford, Oxford, UK; 5 Consorzio Interuniversitario Biotecnologie (CIB), Unit of Brescia, Brescia, Italy

**Keywords:** Bone metastases, Mechanisms of disease, Proteins

## Abstract

Excessive bone disruption, driven by upregulation of bone-degrading osteoclasts, occurs in several pathologies, including breast cancer osteolytic bone metastasis, a condition associated with poor prognosis and diminished quality of life for patients. The matricellular protein thrombospondin-1 (TSP-1) plays pleiotropic roles in physiological and pathological remodeling of several tissues, including bone, and in shaping the microenvironment of primary tumors and metastasis. This study aimed to explore the role of TSP-1 in bone remodeling associated with osteolytic bone metastasis. We have identified a C-terminal fragment of TSP-1, E123CaG, that inhibited RANKL-induced osteoclast differentiation. Cleavage of TSP-1 by serine proteases released by mature osteoclasts, particularly HTRA1, generated a similar fragment, indicating a possible role as a feedback mechanism of control. E123CaG bound RANK, the RANKL receptor on osteoclast precursors, and impaired early (the MAPKs p38 and JNK) and late (NFATc1) downstream signaling. E123CaG also bound osteoprotegerin (OPG), the decoy receptor of RANKL, in this case further potentiating its inhibitory activity by protecting it from degradation by proteases, including HTRA1. In an in vivo model of osteolytic bone metastasis, the expression of E123CaG by murine breast cancer cells reduced osteolytic lesions and prolonged survival, indicating that the C-terminal TSP-1 fragment is also active in vivo and can protect the bone against metastasis-associated osteolysis. Our findings indicate that the release in the bone environment of this TSP-1 fragment, with its unique dual ability to inhibit RANK signaling while potentiating OPG activity, represents an important mechanism to control bone remodeling in osteolytic bone metastasis.

## Introduction

Bones undergo a continuous process of controlled remodeling, where the coordinated activity of bone-degrading osteoclasts and bone-depositing osteoblasts ensures the balance between bone degradation and replacement. Impairment of this balance occurs in several pathologies, including primary and secondary cancers, since the bone is a common site for metastasis, particularly from breast, prostate, and lung cancer [1, 2]. Bone metastases represent a significant clinical challenge, as they are linked to poor prognosis and a diminished quality of life due to complications such as pain, hypercalcemia, and bone fractures.

The mutual interaction of the disseminated cancer cells with resident bone cells leads to the subversion of bone homeostasis and the generation of a tumor-supporting microenvironment [3–6]. In breast cancer metastases, this perturbation of the homeostatic balance commonly results in the formation of osteolytic lesions, characterized by excessive bone degradation. The formation of the lytic lesions is not a direct effect of the tumor cells but is primarily mediated by the deregulated differentiation of osteoclasts in response to different mechanisms that ultimately lead to activation of the RANK/RANKL axis, the main pathway controlling osteoclastogenesis [1, 5]. The receptor activator of nuclear factor-kB ligand (RANKL), mostly expressed by osteoblasts and osteocytes as a transmembrane or soluble protein, triggers the process of differentiation of monocyte precursors into osteoclasts through the interaction with the receptor activator of nuclear factor-kB (RANK). RANK activation by RANKL involves the receptor trimerization, recruitment of adaptor proteins to the intracellular domain, and activation of downstream signaling pathways, characterized by activation of MAPKs (p38, JNK, ERK) and amplification and nuclear translocation of NFATc1, a master regulator of RANKL-induced osteoclast differentiation [7].

Osteoclastogenesis is strictly controlled by the endogenous inhibitor osteoprotegerin (OPG), which acts as a decoy receptor for RANKL. Only 34% of the amino acid sequence is identical between OPG and RANK, but the two proteins share a similar structure in the region containing the four Cysteine-Rich Domains (CRD), of which CRD2 and CRD3 are involved in RANKL recognition [8].

In line with their role in tissue remodeling processes under physiological and pathological conditions, the matricellular proteins thrombospondins (TSPs) have also been implicated in regulating bone remodeling [9, 10]. Mice lacking TSP-1 presented bone abnormalities, including mild spinal deformation and abnormal growth plate organization [11]. In these mice, an increment of bone mass and trabecular and cortical bone volume [12] has been associated with osteoclast functional deficit, as TSP-1 has been reported to promote osteoclastogenesis and bone resorption, mainly mediated by CD36, CD47, TGF-β, integrins, and nitric oxide signaling. On the other hand, TSP-1 can act as a negative regulator of osteoblast maturation, an activity mainly associated with TSP-1 ability to activate latent TGF-β that stimulates the proliferation of osteoprogenitors while inhibiting their late differentiation into osteoblasts. In addition, TSP-1 can control bone remodeling also through other mechanisms, including the control of the composition, structure, and integrity of the extracellular matrix [12, 13].

TSPs are crucial mediators of events associated with cancer progression, including bone cancer and bone metastasis. TSPs, and particularly TSP-1, contribute to shaping the tumor microenvironment and mediate the interaction between the tumor cells and the surrounding environment by acting on the tumor cells but also on other cells, including endothelial cells, fibroblasts, immune cells, and bone cells [14, 15]. The exact role of TSPs in bone metastasis is still to be clarified. Proteomic profiling of bone metastases identified alterations in TSP-1 expression. High levels of TSP-1 protein were found in osteolytic lesions of breast cancer bone metastasis [16] and in multiple myeloma, where TSP-1 ability to activate latent TGF-β contributed to osteolytic bone disease [17], in line with its physiological potentiating activity on bone resorption.

The pleiotropic activities of TSPs in different settings, including bone remodeling and metastasis, are intrinsically linked to its modular structure. The presence of multiple domains and active sites, each interacting with a specific pattern of receptors, soluble factors, matrix components, and proteolytic enzymes, confers dynamic and context-specific roles to these proteins [18]. Factors affecting the bioavailability and activity of the TSP domains, including ligands and the activity of proteases releasing active TSP fragments, determine the final effect in each biological setting. In particular, extracellular proteolytic enzymes can cleave TSP-1, destroying or masking some active sites while increasing the availability of other domains, hence potentially unleashing activities not observed in the intact molecule [19]. In this context, it is noteworthy that mature osteoclasts produce several proteases involved in bone matrix degradation, including proteases known to process TSP-1, such as the serine peptidase HTRA1 and several members of the matrix metalloproteases (MMP) family, such as MMP-14 [20, 21].

Based on these premises, this study was designed to investigate the role of TSP-1 in bone remodeling and osteolytic bone metastasis, focusing on the activity of TSP-1 fragments potentially generated in the protease-rich osteolytic environment, and analyzing their impact on osteoclast differentiation and osteolytic bone metastasis, and their possible interaction with key mediators of osteoclast differentiation (RANKL/RANK/OPG).

## Results

### Effect of TSP-1 and E123CaG on osteoclastogenesis

**Fig. 1:**
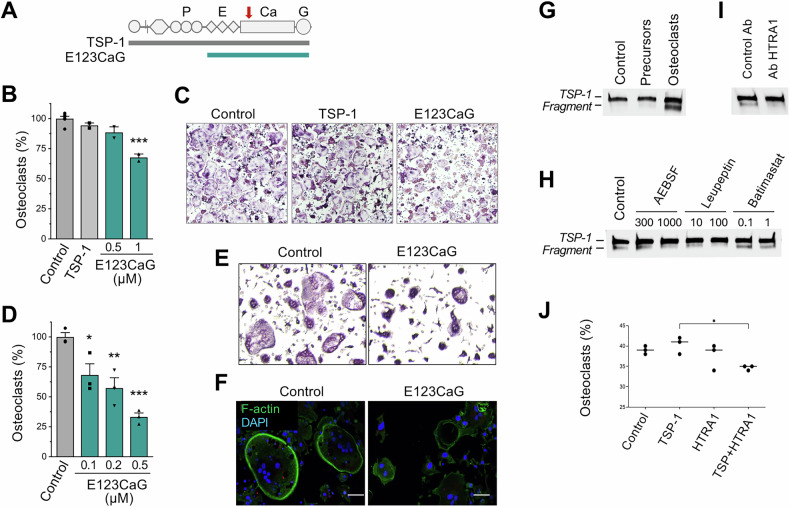
Inhibition of osteoclastogenesis by the TSP-1 fragment E123CaG. **A** Representation of the modular structure of the TSP-1 monomer and the recombinant fragment E123CaG. P, properdin-like type 1 repeats, comprising the binding sites for latent TGF-β and CD36, E, EGF-like type 2 repeats, Ca, calcium-binding type 3 repeats interacting with β3 integrins, and G, globular C-terminal domain binding to CD47. Arrow, epitope of A6.1 antibody in the first calcium-binding motif. **B**, **C** Effect of TSP-1 and E123CaG on RAW 264.7 differentiation. Cells were exposed to RANK-L (50 ng/ml), TSP-1 (1 µM), or the recombinant fragment (0.5 and 1 µM), added on day 0 and day 2. Osteoclasts, identified as TRAP+ cells with more than three nuclei, were quantified on day 4. **B** The graph shows osteoclasts (percentage of control), mean ± SEM of one experiment representative of five. **C** Representative images of osteoclasts under the indicated condition. **D**–**F** Effect of E123CaG on the differentiation of BM precursors, induced by M-CSF (50 ng/ml) and RANK-L (100 ng/ml). E123CaG was added at the indicated concentration on day 1 and day 3. TRAP+ cells with more than three nuclei were quantified on day 5. **D** Number of osteoclasts (percentage of control), mean ± SEM of triplicates from one experiment representative of three. **E** Representative images of osteoclasts in control conditions or in the presence of E123CaG (0.5 µM). *P < 0.05, **P < 0.01, ***P < 0.0001, one-way ANOVA and Tukey’s. **F** Formation of the actin ring in mature osteoclasts in control conditions and in the presence of E123CaG (0.5 µM). Scale bar = 50 µm. **G**–**I** Proteolytic processing of TSP-1 by osteoclasts. **G** TSP-1 (0.5 μg/well) was added to RAW 264.7 cells previously exposed (osteoclasts) or not (precursors) to RANKL. After 24 h, conditioned media were collected and analyzed by WB (Control, TSP-1 not exposed to cells). **H** Effect of protease inhibitors, added at the indicated concentration (in µM) on TSP-1 degradation by mature osteoclasts (Control, TSP-1 exposed to osteoclasts in the absence of inhibitors). **I** Effect of anti-HTRA1 or control antibodies (20 µg/ml) on TSP-1 degradation by mature osteoclasts. **J** Effect of exogenously added HTRA1 (10 µg/ml), with or without TSP-1 (0.4 µM), on osteoclastogenesis (*P < 0.05, one-way ANOVA and Tukey’s).

Modulation of osteoclastogenesis by TSP-1 has been mainly ascribed to its interaction with TGF-β, CD36, CD47, growth factors, and integrins. Using RAW 264.7, a monocyte/macrophage-like cell line used as a conventional model of osteoclast differentiation [22, 23], we investigated the activity on osteoclastogenesis of the entire TSP-1 protein and the recombinant fragment E123CaG, lacking the binding site for TGF-β and CD36 but retaining the capacity to interact with growth factors, CD47, and integrins (Fig. 1A).

In the experimental conditions adopted, the differentiation of RAW 264.7 cells induced by RANKL was not affected by TSP-1. It was instead inhibited by the fragment E123CaG that reduced the number and size of mature, TRAP-positive osteoclasts (Fig. 1B, C). The lack of the expected stimulatory activity of TSP-1 was likely due to the prevailing action of RANKL, used at concentrations sufficient to induce the maximum possible differentiation response, hence overpowering the possible effect of the TSP-1/CD36 and TSP-1/TGF-β interactions. E123CaG did not affect the proliferation of undifferentiated RAW 264.7 cells (not shown).

E123CaG inhibited, even more potently, the differentiation of murine bone marrow (BM) precursors induced by M-CSF and RANKL, confirming its ability to affect osteoclastogenesis also in a more physiological system (Fig. 1D, E). In this system, E123CaG reduced the number and size of osteoclasts and also affected the cytoskeleton reorganization associated with osteoclast maturation (Fig. 1F), inhibiting the formation of the actin ring, which defines the sealing zone of mature, active osteoclasts [24].

### Proteolytic processing of TSP-1 by osteoclasts

The activity of TSPs is controlled by extracellular proteolytic processing, which regulates the bioavailability of the different active sites in a context-specific manner [19]. We found that mature osteoclasts, but not undifferentiated precursors, proteolytically processed TSP-1, releasing one major proteolytic fragment likely corresponding to E123CaG, since it was recognized in WB by the A6.1 antibody directed against the C-terminal portion of TSP-1 [25] (Fig. 1A, G). Proteolysis of TSP-1 was prevented by the inhibitors of serine proteases leupeptin and AEBSF, but not by the broad-spectrum inhibitor of matrix metalloproteases batimastat (Fig. 1H), pointing to the involvement of serine proteases in this process. Among the serine proteases released by osteoclasts, HTRA1 is able to degrade TSP-1 [26–28] (Supplementary Fig. 1A). We found that TSP-1 degradation by osteoclasts was prevented by antibodies against HTRA1 (Fig. 1I), whereas, in line with the lack of activity of batimastat, antibodies against MMP-14 were not effective (Supplementary Fig. 1B). Moreover, when TSP-1 was added to the RAW 264.7 differentiation assay in the presence of exogenous HTRA1, inhibition of osteoclastogenesis was observed, confirming that HTRA1 can indeed release the inhibitory fragment from TSP-1 (Fig. 1J). These findings indicate that serine proteases released by mature osteoclasts, and particularly HTRA1, can process TSP-1 in the bone microenvironment. The released fragment, comprising E123CaG, might represent a physiological feedback mechanism to control osteoclast differentiation and prevent excessive bone degradation.

### E123CaG binds RANK

**Fig. 2:**
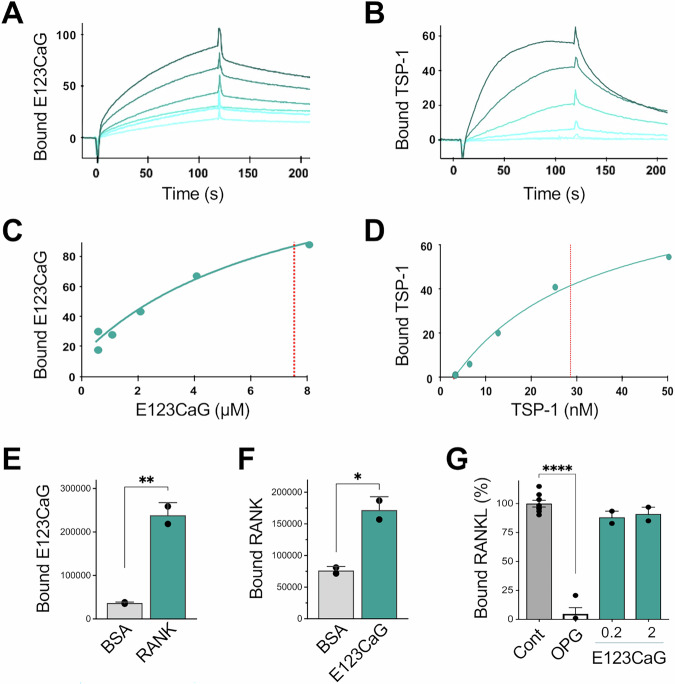
Interaction of E123CaG with RANK. **A**, **B** SPR blank-subtracted sensorgram overlay showing the binding of increasing concentrations of E123CaG (**A**) or TSP-1 (**B**) to RANK-coated sensor chip. **C**, **D** SPR saturation curve obtained using the values of RU bound at equilibrium from injection of the increasing concentrations of E123CaG (**C**) or TSP-1 (**D**) onto immobilized RANK. The amount of bound molecules are expressed in RU. Results are from one experiment representative of four (E123CaG) or three (TSP-1). **E**, **F** Solid phase analyses of the binding of biotinylated E123CaG (45 nM) to immobilized RANK (**E**), and binding of biotinylated RANK (50 nM) to immobilized E123CaG (**F**). Wells saturated with BSA were used as the control. Data are expressed as absorbance, mean, and SD of values from one experiment representative of at least two. *P < 0.05; **P < 0.005, *t* test. **G** Effect of E123CaG on RANKL/RANK interaction. Binding of biotinylated RANKL (15 nM) to immobilized RANK in the presence of the indicated concentration of E123CaG (in µM) or OPG (12 µg/ml). Data are the percentage of control binding (mean ± SD of values from one experiment representative of two). ****P < 0.0001, one-way ANOVA and Dunnett’s.

Given the distinctive ability of TSP-1 domains to bind and modulate the activity of several proteins and cell receptors, we investigated whether the E123CaG fragment might interact with RANK, the receptor of RANKL on osteoclasts. SPR was first conducted to evaluate the interaction of E123CaG with RANK and calculate the binding affinity. E123CaG bound RANK in a dose-dependent manner (Fig. 2A, C), with a dissociation constant (*Kd*) in the micromolar range (*Kd* = 6.3 ± 2.6 μM). The entire TSP-1 also bound immobilized RANK (Fig. 2B, D) and with a higher affinity compared to E123CaG (*Kd* = 0.026 ± 0.015 µM), a finding probably due to the trimeric nature of TSP-1 with a consequent, possible avidity affect (see “Discussion”).

E123CaG interaction with RANK was observed also in solid phase binding assays, which showed that labeled E123CaG bound immobilized RANK (Fig. 2E) and, vice versa, that biotinylated RANK bound immobilized E123CaG (Fig. 2F).

### E123CaG does not affect RANKL binding to RANK

We then investigated whether E123CaG, by binding RANK, might interfere with the RANKL/RANK interaction. In a solid phase competition assay, E123CaG did not reduce the ability of RANKL to bind immobilized RANK (Fig. 2G). Binding was instead inhibited by OPG, used as a reference inhibitor, although with a different competition mechanism (as its binds RANKL, while E123CaG binds RANK). Similar results were obtained using a commercial RANK/RANKL binding assay kit (RayBio), where E123CaG did not compete for the binding of unlabeled RANKL to RANK (Supplementary Fig. 2A). Moreover, E123CaG did not prevent the binding of Europium-labeled RANKL to RAW 264.7 cells (Supplementary Fig. 2B), confirming the lack of binding competition also in a more physiological setting that preserves the receptor plasticity and the physiological movements in the cell membrane required for its activation.

### E123CaG counteracts RANKL-RANK signaling

**Fig. 3:**
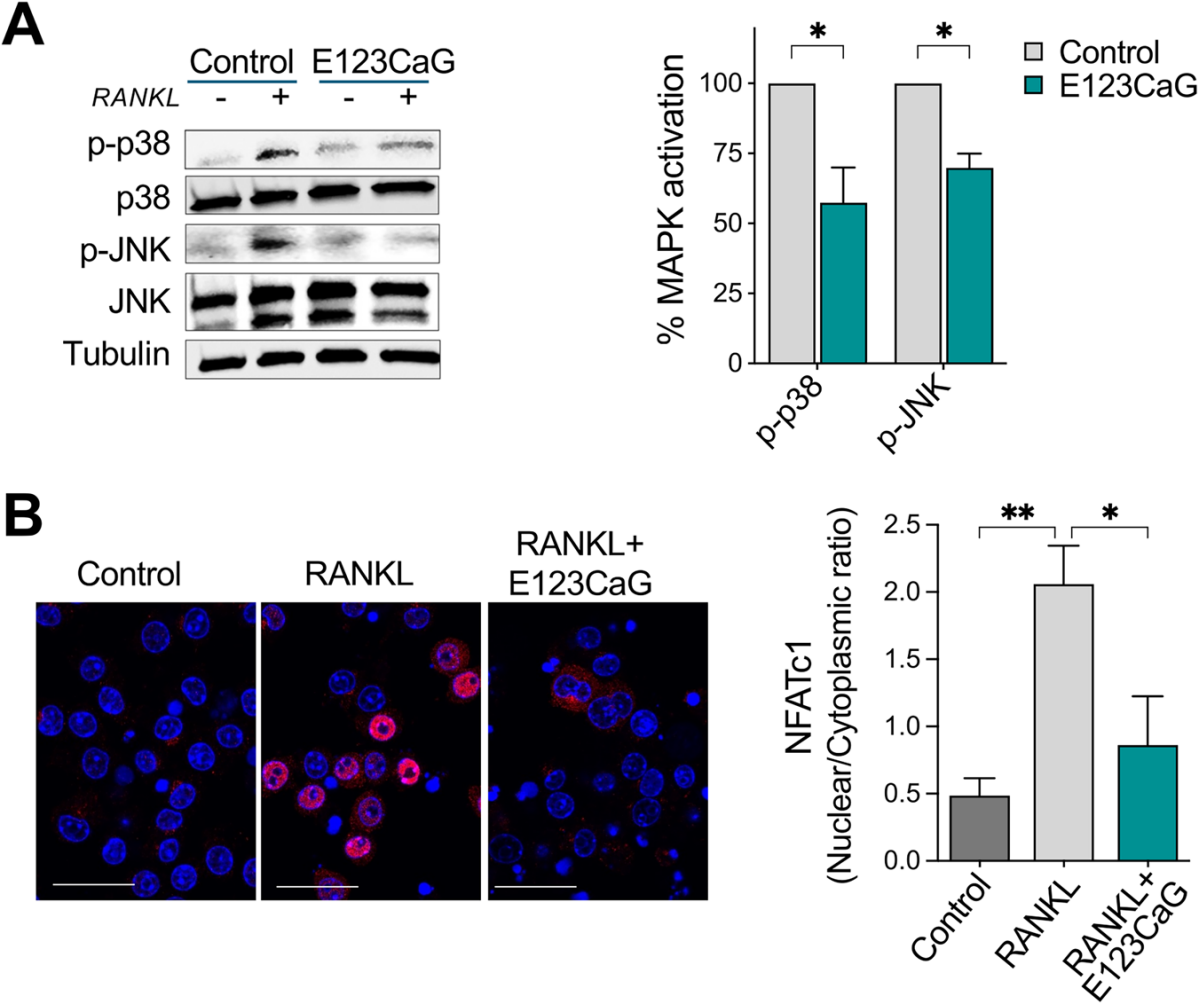
Effect of E123CaG on early and late RANK signaling. RAW 264.7 cells, pretreated with E123CaG (1 μM) in serum-free medium for 3 h were stimulated with RANKL (50 ng/ml) for 20 min (**A**) or 48 h (**B**). **A** Western blot analysis of phosphorylated p38 (p-38), total p38, phosphorylated JNK (p-JNK), and total JNK. Tubulin was used as a loading control. Graph, quantitative analysis. MAPK activation (phosphorylated protein/total protein ratio) is expressed as the percentage of controls, mean ± SEM of two independent experiments (*P < 0.05 *t* test). **B** Nuclear translocation of NFATc1. Representative images of cells treated (RANKL) or not (Control) with RANKL with or without E123CaG (blue, nuclei; red, NFATc1, scale bar 25 µm). Graph, NFATc1 distribution is expressed as a nuclear to cytoplasmic ratio. Data are mean ± SEM from one experiment representative of two (**P < 0.002; *P < 0.05 one-way ANOVA and Tukey’s).

We next investigated whether E123CaG, although not interfering with the RANKL/RANK interaction, might affect RANK’s signal transduction capability. Exposure of RAW 264.7 cells to RANKL induced the expected signaling activation involving an early response, characterized by MAPK activation, followed by the increased expression and nuclear translocation of NFATc1, the main transcription factor responsible for the transcription of genes necessary for osteoclast differentiation [29–31]. The presence of E123CaG reduced the early signaling events, inhibiting MAPKs activation, with a significant reduction of p38 and JNK phosphorylation (Fig. 3A). E123CaG also counteracted the upregulation of NFATc1 and reduced its translocation from the cytosol to the nucleus, indicating its inhibitory activity also on the late signaling events of the RANKL/RANK cascade (48 h after RANKL exposure) (Fig. 3B).

All together, these results demonstrate that E123CaG binding to RANK does not prevent RANK binding to the endogenous ligand RANKL, but impairs RANK downstream signaling cascade.

### E123CaG binds OPG

**Fig. 4:**
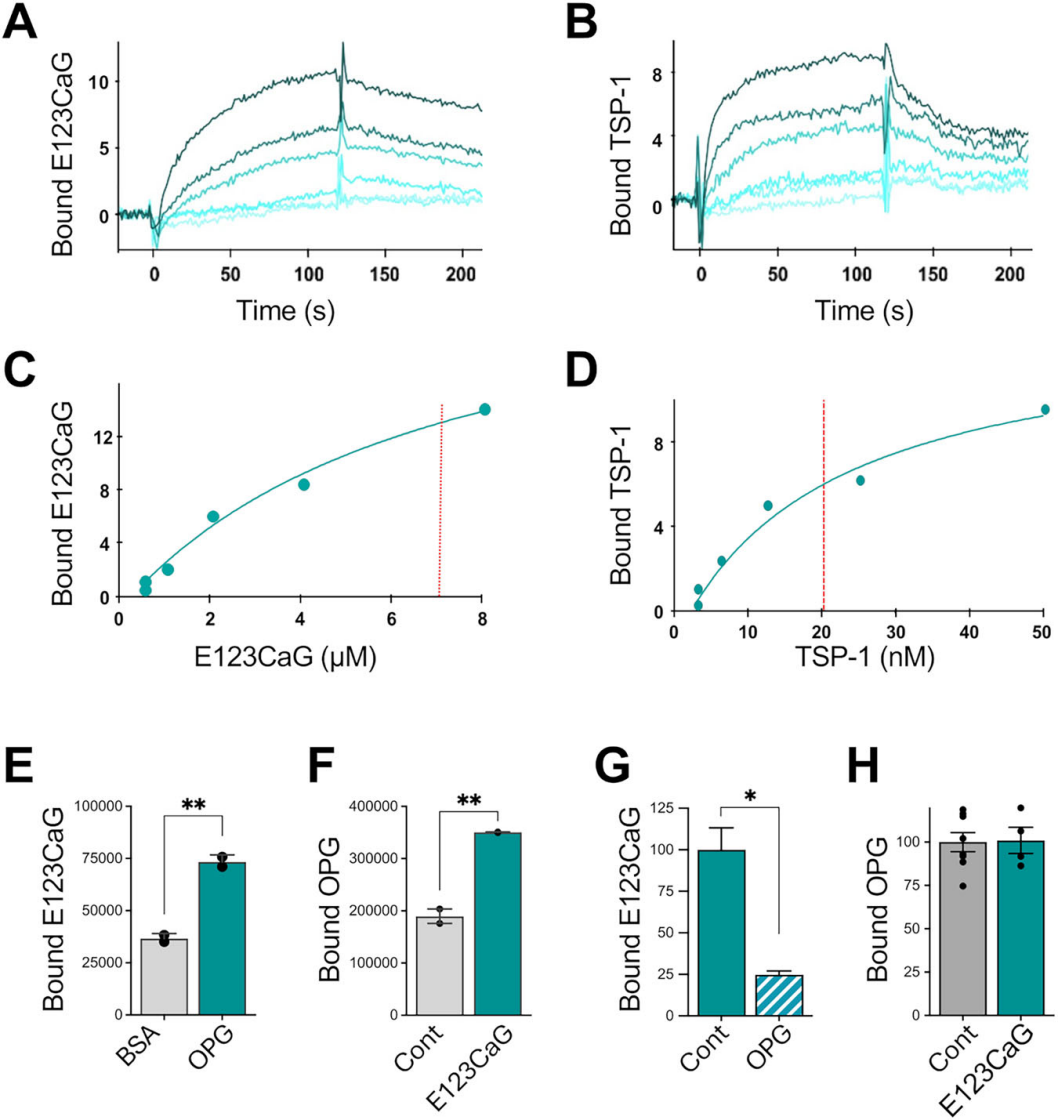
Interaction of E123CaG with OPG. **A**, **B** SPR blank-subtracted sensorgram overlay showing the binding of increasing concentrations of E123CaG (**A**) or TSP-1 (**B**) to OPG-coated sensor chip. **C**, **D** SPR saturation curve obtained using the values of RU bound at equilibrium from injection of the increasing concentrations of E123CaG (**C**) or TSP-1 (**D**) onto immobilized OPG. The amount of bound molecules is expressed in RU. Results are from one experiment representative of five (E123CaG) or six (TSP-1). **E**, **F** Solid phase analyses of the binding of biotinylated E123CaG (45 nM) to immobilized OPG (**E**), and binding of biotinylated OPG (50 nM) to immobilized E123CaG (**F**). Wells saturated with BSA were used as the control. Data are expressed as absorbance, mean, and SD of values from one experiment representative of at least two. **P < 0.01, *t* test. **G** Binding of biotinylated E123CaG (15 nM) to immobilized RANK in the presence of OPG (200 nM). **H** Effect of E123CaG on OPG/RANKL interaction. Binding of biotinylated OPG to immobilized RANKL in the presence of E123CaG. In G and H, data are the percentage of control-specific binding, mean ± SEM of values from one experiment representative of two (*P < 0.05, *t* test).

Given the structural similarity with RANK, we investigated the possible binding of E123CaG with another member of the TNFR superfamily, the physiological inhibitor OPG, that acts as a decoy receptor for RANKL. Only 34% of the sequences of OPG and RANK are identical, but they share a similar structure in the RANKL-recognizing cysteine-rich domains [8]. In SPR analysis, E123CaG bound OPG (Fig. 4A, C), with an affinity similar to that of its interaction with RANK, as indicated by the comparable dissociation constants (*Kd* = 7.5 ± 0.5 μM). As in the case of RANK, also the entire TSP-1 was able to bind OPG, and with a higher affinity compared to E123CaG (Kd = 0.020 ± 0.019 μM, Fig. 4B, D). In solid phase assays, we found that labeled E123CaG bound immobilized OPG (Fig. 4E) and that biotinylated OPG bound immobilized E123CaG (Fig. 4F). In this system, OPG reduced the interaction of the TSP-1 fragment with RANK (Fig. 4G), suggesting that the E123CaG binding regions for the two ligands somewhat overlap. Conversely, E123CaG did not impair OPG binding to its physiological substrate, RANKL (Fig. 4H), as also occurred with RANK, indicating that E123CaG does not bind the RANKL-interacting site of either RANK or OPG.

### E123CaG potentiates the inhibitory activity of OPG on osteoclastogenesis

**Fig. 5:**
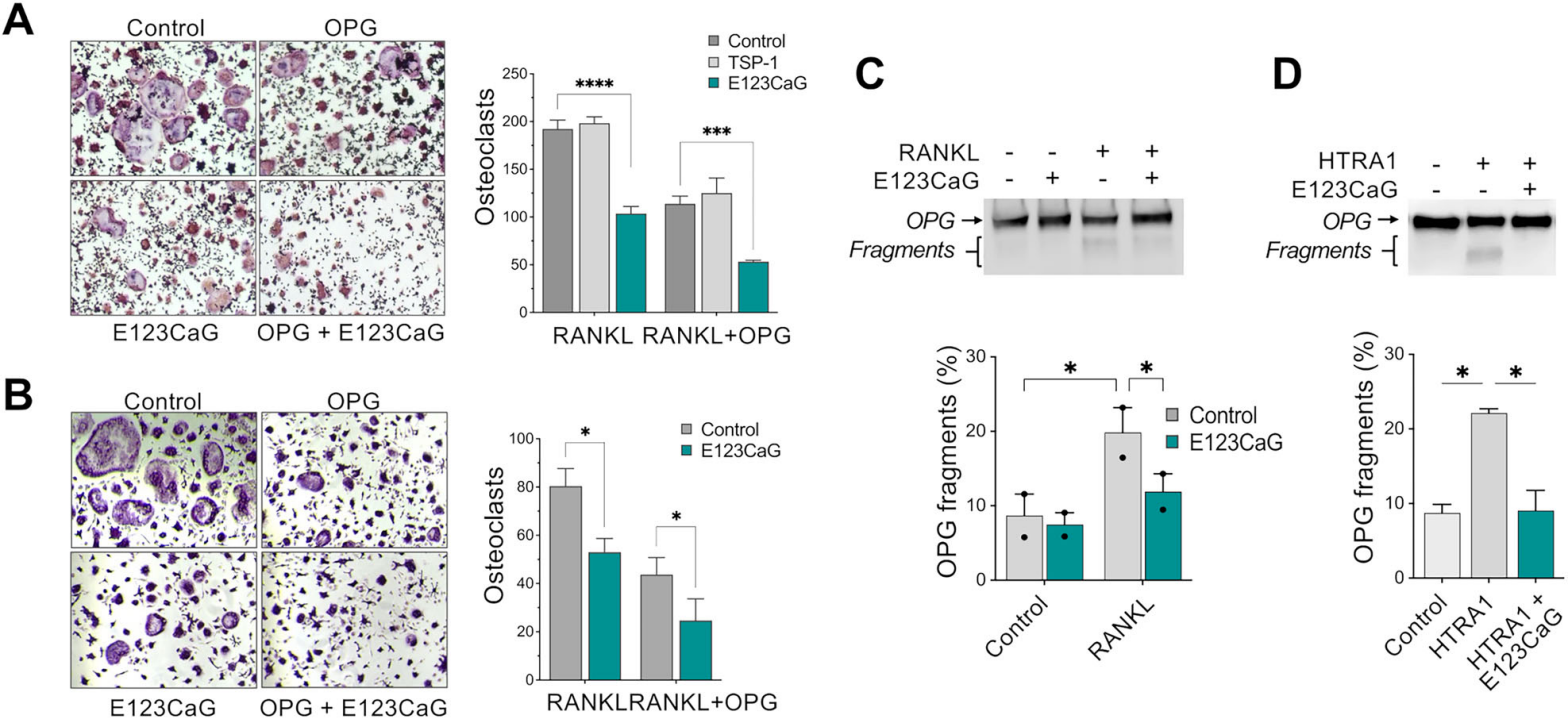
E123CaG potentiates the inhibitory effect of OPG. **A** RAW 264.7 cells were induced to differentiate into osteoclasts by RANK-L (50 ng/ml) in the presence or not of OPG (30 ng/ml), TSP-1, or E123CaG (1 µM), all factors added on day 0 and 2. TRAP+ multinucleated cells (more than three nuclei) were quantified on day 4. **B** BM-derived precursors were induced to differentiate into osteoclasts by M-CSF (50 ng/ml) and RANKL (100 ng/ml) in the presence or not of OPG (60 ng/ml) and E123CaG (0.5 µM), added on days 1 and 3. Osteoclasts were counted on day 5. Graphs show the number of osteoclasts, mean ± SEM of triplicates, from one experiment representative of three (two-way ANOVA and Tukey’s, *P < 0.05, **P < 0.001, ***P < 0.0001). **C** Proteolytic degradation of OPG. RAW 264.7 cells were exposed or not to RANKL to induce osteoclastogenesis. Biotinylated OPG (0.15 μg/well) was added to mature osteoclasts in serum-free medium, in the presence or not of E123CaG (1 µM). **D** Degradation of biotinylated OPG (0.1 µg) exposed to recombinant HTRA1 (0.1 µg) in the presence or absence of E123CaG (1 µM). **C**, **D** After a 24 h incubation, the formation of OPG fragments was evaluated by WB, with ExtrAvidin−Peroxidase. The graphs show OPG fragmentation, expressed as the percentage of fragments over total OPG (one experiment representative of two).

We next investigated whether E123CaG might affect OPG activity as an inhibitor of RANKL-induced osteoclastogenesis. OPG was used at a suboptimal concentration, allowing for both an increase and a decrease in the inhibitory effect to be observed. E123CaG not only did not diminish, but actually potentiated OPG inhibition of RANKL-induced differentiation of RAW 264.7 (Fig. 5A) or of murine bone marrow precursors (Fig. 5B). TSP-1, although also able to bind OPG, had no effect at equimolar concentrations Fig. 5A).

### E123CaG protects OPG from proteolytic degradation

OPG is degraded during osteoclastogenesis by proteases released by mature osteoclasts, particularly by HTRA1, conceivably as a mechanism to control its inhibitory activity [20]. Since TSP-1 has been reported to protect soluble factors from proteolytic degradation [32] and inhibit the activity of proteases, including HTRA1 [27], we investigated whether E123CaG might prevent OPG degradation.

We found that mature osteoclasts, but not undifferentiated precursors, degraded OPG, and proteolysis was reduced by E123CaG (Fig. 5C). In addition, E123CaG protected OPG from proteolytic degradation by recombinant HTRA1 (Fig. 5D). These findings indicate the ability of E123CaG to protect OPG against osteoclast-mediated degradation, prolonging OPG bioavailability, thus contributing to the osteoclast inhibitory activity of the TSP-1 fragment.

### Protective activity of E123CaG in a model of osteolytic bone metastasis

4T1.2 is a well-characterized murine breast cancer cell line, syngeneic in BALB/c mice, commonly used as a model of highly metastatic breast cancer with a strong propensity to metastasize to the bone [33, 34]. To study the effect of E123CaG on osteolytic bone metastasis, 4T1.2 tumor cells were engineered to express and secrete the TSP-1 fragment, hence obtaining a continuous, local release of the protein, adopting the approach previously used to study other TSP-1 fragments [35]. Control cells were transfected with the empty vector (Supplementary Fig. 3A, B). The production of E123CaG did not alter tumor cell proliferation in vitro (Suppl. Figure C) and did not affect the growth of orthotopic tumors in vivo, after implantation in the mammary fat pad (mfp, Supplementary Fig. 4). Despite the selection for bone tropism, the injection of 4T1.2 into the mfp generated spontaneous metastasis mainly to the lungs with a low rate of bone metastasis, making this orthotopic system not suitable to study the effect of E123CaG on bone metastasis. We therefore opted for the more reliable model of artificial bone metastasis, obtained by injecting tumor cells in the caudal artery (CA) of mice [36, 37]. CA injection preferentially directs the injected tumor cells to the hind limbs, resulting in the development of sizeable osteolytic bone metastasis in tibiae and femurs. The development of bone metastasis produced extensive damage in the epiphysis and metaphysis of the proximal tibia and distal femur, detectable by micro-CT two weeks after cell injection, and profoundly destroying the trabecular bone (Supplementary Fig. 5).

**Fig. 6:**
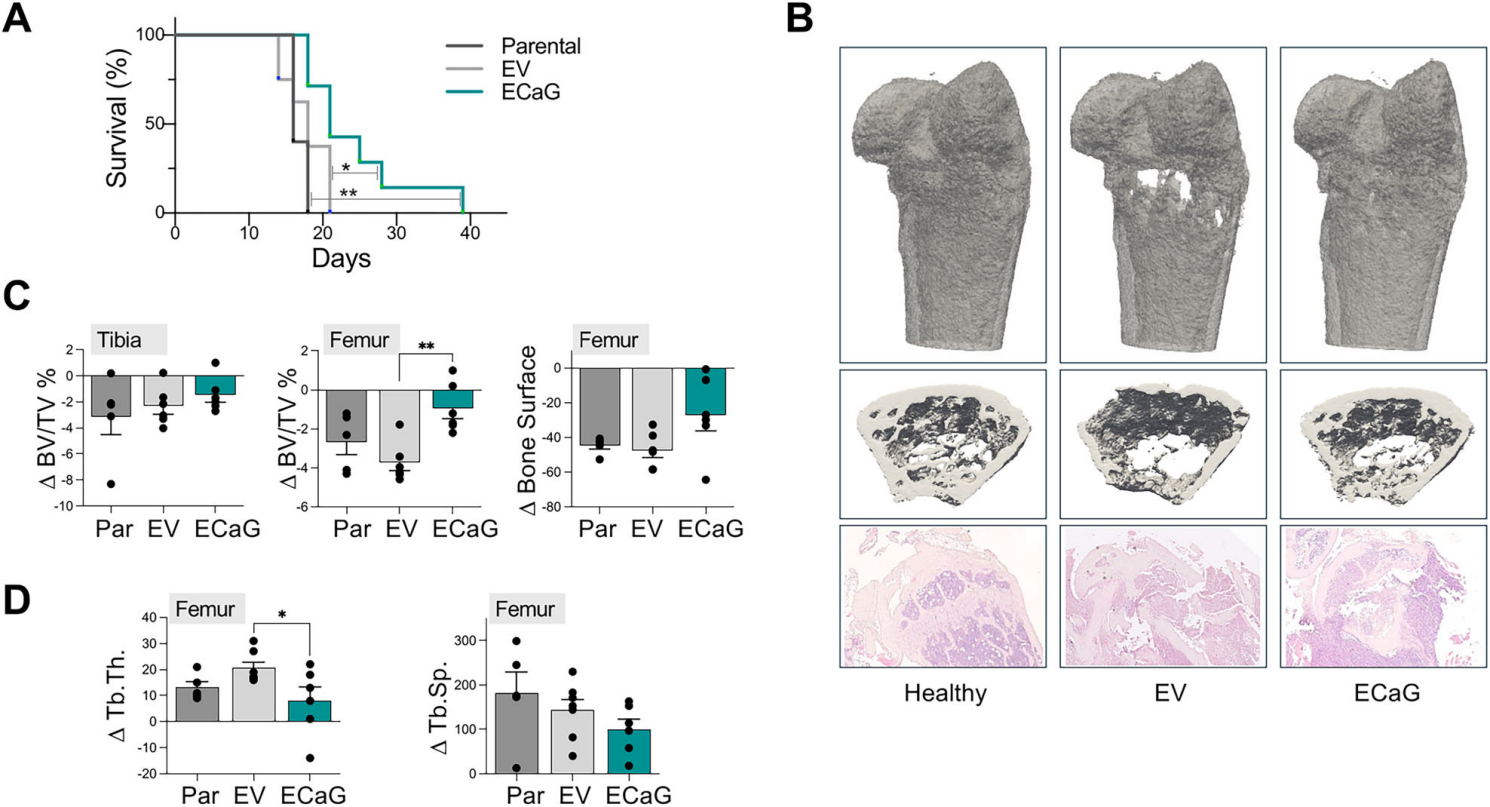
Effect of E123CaG on the 4T1.2 in vivo model of osteolytic bone metastasis. 4T1.2 cells (10^5^/mouse), parental (4T1.2 Parental, Par, *n* = 5) or control 4T1.2 EV transfected with the empty vector (EV, *n* = 8) or the E123CaG-expressing 4T1.2 E123CaG (ECaG, *n* = 7) were injected into the caudal artery of mice. **A** Survival of mice. Survival (expressed in %) refers to the time of suppression at humane endpoints (days after tumor cell injection) (Log-rank test, *P = 0.03, **P = 0.005). **B**–**D** Micro-CT analysis of bone degradation. Mice injected with the parental 4T1.2 cells, 4T1.2 EV or 4T1.2 E123CaG were monitored for bone damage by micro-CT on day 0 and 14. **B** Representative three-dimensional reconstructions of femurs (top panels) and trabecular areas (middle panels), and histological analysis of tibias (H&E, bottom panels) in a healthy mouse and 14 days after CA tumor cell injection, showing evident osteolytic lesions in 4T1.2 EV and the protective effect of E123CaG. **C**, **D** Bone structural parameters were calculated using the BoneJ plugin of ImageJ. **C** BV/TV% and bone surface were analyzed in selected ROIs (500 ×9 µm) covering the proximal tibia and distal femur epiphysis. **D** Quantitative analysis of trabecular bone mean thickness (Tb.Th.) and separation (Tb.Sp.) analyzed in 150 slices covering the trabecular area of the distal femur (slices 255–404 of the initial 500-slice selection). All data are expressed as the difference between day 14 and day 0 values. *P < 0.05, **<0.05p One-way ANOVA, followed by Tukey’s multiple comparison test.

After CA injection, parental 4T1.2 cells and control cells transfected with the empty vector (EV group) exhibited a similar progression of the disease over time, with comparable survival curves. In contrast, the expression and secretion of E123CaG by tumor cells (ECaG group) significantly prolonged the survival of mice, delaying the onset of bone metastasis (Fig. 6A).

To evaluate bone degradation and the formation of osteolytic lesions, micro-CT scans of hind limbs were performed before and two weeks after the injection of parental, control, and E123CaG-expressing 4T1.2 cells. Analysis focused on the bone areas mostly affected by metastasis, selecting ROIs in 500, 9 μm thick slices (i.e., 4.5 mm) starting from the head of epiphysis of distal femur and proximal tibia. E123CaG significantly counteracted bone degradation in the hind limbs, with a major effect in the distal femur, as indicated by the reduction in the bone volume fraction (BV/TV) and bone surface loss (Fig. 6B, C). The production of E123CaG led to a diminished degradation of the trabecular bone (slices 255–404 of the initial 500-slice selection), with increased trabeculae thickness and reduced space between adjacent trabeculae (Fig. 6B, D).

All together, these results highlight the inhibitory effect of E123CaG on bone degradation in osteolytic bone metastasis in vivo, in line with the effect on osteoclastogenesis shown in vitro.

## Discussion

The complex molecular mechanisms governing the interplay between tumor cells and the bone microenvironment, which are responsible for metastasis-associated bone alterations, are still not completely clarified. This study has identified a fragment of the matricellular protein TSP-1, E123CaG, as a novel inhibitor of osteoclast differentiation, possibly acting as a physiological feedback mechanism to control the process of bone remodeling, and able to reduce bone degradation in a model of breast cancer osteolytic metastasis.

The multidomain, modular structure of TSP-1 is responsible for its pleiotropic activities. The presence and accessibility of the various active sites contribute to the complex regulation of tissue remodeling and tumor–microenvironment interactions. The fact that a fragment exhibits biological activity absent in the entire molecule is not uncommon in TSPs and many extracellular matrix proteins, which contain multiple, frequently cryptic active sites that become functional once exposed, in a context-dependent manner. The different activity of the entire TSP-1 and the E123CaG fragment on osteoclast differentiation is mainly attributable to the presence and the different ligand binding affinity of additional active sites on the entire TSP-1, particularly the binding sites for TGF-β and CD36, known to be involved in osteoclast differentiation. In addition, different ligands as well as the presence of adjacent domains can control the exposure of active sites and affect TSP-1 local conformation, thus contributing to the different activity of the two molecules. Finally, the trimeric nature of TSP-1 as opposed to the monomeric form of the fragment can profoundly influence the composition and organization of multimolecular complex networks orchestrated by TSP-1 in the pericellular space.

Proteolytic degradation of TSP-1 is a critical mechanism for the control of protein activity, through the release of active fragments. Different extracellular proteases cleave TSPs at specific sites. Consequently, the nature and activity of the TSP fragments generated within a particular biological environment are determined by the type and abundance of local proteases. This interplay thus provides a robust mechanism for modulating TSP activity and ensures a rapid adaptive response to microenvironmental changes.

Differentiated osteoclasts produce proteases involved in bone-resorbing activity. Among the most expressed proteases, the membrane-type MMP14 (MT1-MMP) and the serine protease HTRA1 can process TSP-1 [21, 26–28]. Our findings with selective protease inhibitors and antibodies indicate a major role for HTRA1 in TSP-1 cleavage by osteoclasts, whereas MMPs, specifically MMP-14, are apparently not involved. We cannot however rule out the possible role of other TSP-1-degrading proteases produced by osteoclasts, such as the serine protease KLK4, previously reported able to degrade TSP-1 in prostate cancer bone metastases [38].

E123CaG is a large fragment of TSP-1 and contains binding sites for several ligands, including CD47, integrins, and growth factors, reported to be active in osteclastogenesis. The involvement of CD47 in the mechanism of E123CaG inhibition is unlikely, since CD47 has been involved in the stimulating activity of TSP-1 on osteoclast differentiation through the promotion of cell fusion [39] and inhibition of nitric oxide signaling [40]. On the other hand, our findings indicate that the presence of the CD47 interaction sequence did not prevent the inhibitory activity of E123CaG.

We therefore focused our research on other potential mediators of E123CaG activity on osteoclasts and identified the receptor RANK and the decoy receptor OPG as new, hitherto undescribed, ligands of TSP-1.

Both E123CaG and TSP-1 bind RANK. The higher affinity of TSP-1 (lower Kd) is in line with previous findings comparing the affinity of TSP-1/TSP-2 and their active fragments for FGF-2 [41, 42] and might depend on the known influence of flanking domains on the structure, activity, and ligand binding affinity of TSP-1 active sites [43]. Moreover, we cannot rule out the possibility that the trimeric structure of the native TSP-1—as opposed to monomeric E123CaG—might cause an apparent slower dissociation rate due to an avidity effect, as reported [44].

The binding of E123CaG to RANK did not impair the receptor ability to bind RANKL but significantly affected RANK early and late signaling, reducing activation of the MAPK cascade and impairing nuclear translocation of the transcription factor NFATc1, a master regulator of osteoclast differentiation. The mechanism of inhibition of RANK signaling by E123CaG is still unclear. RANK trimerization following RANKL binding is required to trigger the activation of the downstream osteoclastogenic signaling pathway and impairment of trimer formation prevents the signaling cascade and inhibits osteoclastogenesis [45]. The possibility that inhibition of RANK trimerization is a mechanism of E123CaG inhibition of osteoclastogenesis warrants further investigation.

Our findings indicate that E123CaG also binds OPG. Similarly to the RANK/RANKL interaction, E123CaG did not prevent the binding of OPG to RANKL, however, and in contrast to RANK, it did not impair OPG activity but actually enhanced the inhibitory effect of OPG on osteoclastogenesis.

This augmented inhibition could be due to an additive effect of the two molecules acting independently. On the other hand, the ability of E123CaG to bind OPG points to a potential effect on OPG activity and/or bioavailability. In agreement, we found that degradation of OPG by either osteoclasts or HTRA1 was prevented by E123CaG. TSPs are known modulators of protease activity, by inhibiting the activity of proteases or by binding protease substrata, protecting them from degradation [19, 32]. It is therefore possible that the protective activity of E123CaG on OPG and the inhibitory activity on HTRA1 [27] might contribute to the protective effect of the TSP-1 fragment against proteolytic degradation.

**Fig. 7:**
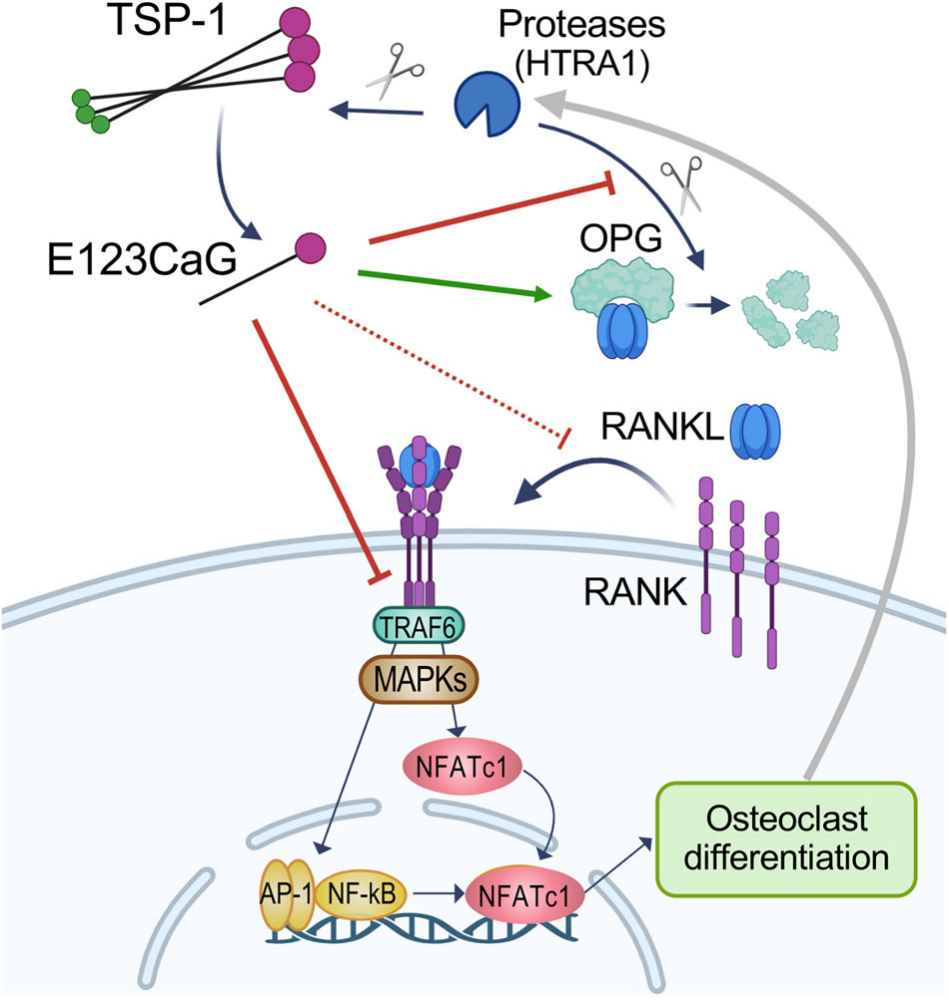
Proposed model of the activity of E123CaG on osteoclastogenesis, and its interactions with RANK, OPG, and HTRA1. E123CaG, released by proteolytic processing of TSP-1 by osteoclast-derived proteases, particularly HTRA1, binds to RANK, impairing its downstream signaling (possibly by affecting its trimerization or signal transduction ability, dotted line). E123CaG also binds OPG and protects it from proteolytic degradation by proteases, including HTRA1, potentiating its inhibitory effect on osteoclastogenesis (green arrow) (image created with Biorender).

All together, our findings point to a complex molecular and functional interplay between TSP-1, HTRA1, and the RANK/RANKL/OPG system in the control of bone remodeling (Fig. 7). HTRA1, produced by mature osteoclasts, can degrade TSP-1 releasing a fragment that, in turn, can inhibit osteoclast differentiation by interacting with RANK and OPG. By interacting with RANK on the surface of osteoclast precursors, E123CaG inhibits RANKL-induced intracellular pathways, reducing MAPKs activation and the nuclear translocation of NFATc1. By interacting with OPG, E123CaG potentiates OPG inhibitory effect on osteoclast differentiation, protecting the inhibitor from proteolytic degradation by proteases, including HTRA1 (Fig. 7).

We cannot exclude the possible involvement of other factors in the activity of E123CaG, and specifically integrins, important players in bone remodeling and osteoclastogenesis. In particular, α_v_β_3_ integrin mediates a co-stimulatory signal associated to RANK/RANKL axis and control osteoclast maturation, adhesion, and the formation of the actin ring necessary for bone resorption [46]. Since E123CaG contains the α_v_β_3_ integrin recognition sequence arginine-glycine-aspartic acid (RGD) in the type 3 repeats (Ca) domain, the involvement of this integrin in E123CaG activity is plausible, in line with the observed impaired formation of the actin ring in the presence of the TSP-1 fragment.

Both RANK and OPG have been reported to be involved in immune response, inflammation, and tumor cell malignant behavior [47–49]. The identification of RANK and OPG as ligands of TSP-1 and E123CaG opens the way to further studies to investigate whether these interactions affect other functions of OPG and RANK in cancer metastasis, beyond the regulation of osteoclastogenesis.

The finding that E123CaG delayed the onset of osteolytic lesions in an in vivo model of breast cancer bone metastasis indicates that the inhibitory effect of the fragment on osteoclastogenesis observed in vitro also occurs in vivo, where E123CaG appears to protect the bone against osteolysis associated with metastasis. The clinical relevance of these findings requires additional investigation, particularly given the complexity of the system and the multiple roles of the molecules involved. Evidence that HTRA1 expression is associated with a positive prognosis in a cohort of 131 breast cancer patients and in publicly available datasets [50] suggests a protective role of the protease against malignant progression. This is further supported by a proteomic analysis, which identified TSP-1 and HTRA1 among the molecules downregulated in bone metastasis compared to the matched primary tumor, indicating that bone metastasis may progress when these protective factors are simultaneously suppressed [51].

Given the variety of activities referable to multiple active sites in E123CaG, it is possible that, besides its effect on osteoclasts, other mechanisms contribute to the antimetastatic effect. We have ruled out a direct effect of the TSP-1 fragment on tumor cell proliferation, since the two tumor variants, expressing or not E123CaG, had the same growth rate in vitro and in vivo following orthotopic implantation. Nevertheless, we cannot rule out the possibility that the domain might affect other functions of the tumor cells themselves or modulate their interaction with other components of the bone tumor microenvironment, including endothelial and immune cells.

In conclusion, this study has established a novel role for TSP-1 as a key regulator of the tumor microenvironment in bone metastasis. Our data indicate that, contrary to the osteoclast-stimulating activity described in literature for the entire TSP-1, the E123CaG fragment, lacking the CD36 and TGF-β binding sequences but able to interact with RANK and OPG, inhibits osteoclastogenesis. Since a similar fragment is released by proteolytic cleavage of TSP-1 by mature osteoclasts, this might represent a feedback mechanism of regulation of the process. The fragment was also active in vivo, confirming its potential as a bone-protective factor against bone degradation associated with osteolytic metastasis and possibly other pathologies characterized by excessive bone degradation.

## Materials and methods

### Proteins and labeling

TSP-1 was purified from thrombin-stimulated human platelets [32]. Recombinant TSP-1 fragments were expressed in insect cells using the baculovirus expression system, as described [35, 52] using vectors kindly provided by D.D. Mosher (University of Wisconsin-Madison, WI).

Recombinant human RANK/TNFRSF11A Fc Chimera and OPG/TNFRSF11B were from R&D Systems (Milan, Italy). Murine recombinant murine RANK-L was from PeproTech (Thermo Fisher Rodano, Italy).

#### Biotin-labeling

E123CaG, RANKL, and OPG were biotinylated with Biotinamidohexanoic acid 3-sulfo-N-hydroxysuccinimide ester sodium salt (Sigma-Aldrich, Merk, Milan, Italy) in PBS at a molar ratio of 15:1 (biotinylation reagent to protein) for 30 min at room temperature. The biotinylated proteins were then isolated by ZebaTM Spin Desalting Columns 7 K MWCO (Thermo Scientific, Segrate, Italy). Labeling was confirmed by western blot analysis, using ExtrAvidin-Peroxidase (Sigma-Aldrich) as described below.

### Solid-phase binding assays

DELFIA microtiter plates and reagents (PerkinElmer, Milan, Italy) were used. Plates were coated overnight at 4 °C with RANK or OPG (0.5 µg/40 µl/well) or RANKL (0.2 µg/40 µl/well) in PBS. After saturation of non-specific binding sites by a 30 min-incubation with 1% BSA in PBS, biotin-labeled E123CaG, RANKL or OPG were added at the indicated concentration in 1% BSA-PBS, in the presence or absence of the indicated competitors, and incubated at room temperature for 3 h. Wells were then washed with PBS 0.1% BSA and incubated for 1 h with Eu-labeled Streptavidin (Sigma, 1:1000) at room temperature. After washing, plates were incubated with DELFIA Enhancement Solution, and bound biotin-labeled ligands were quantified measuring time-resolved fluorescence using a Victor3 multilabel plate reader (PerkinElmer). Specific binding was calculated by subtracting non-specific binding to uncoated, BSA-saturated plastic.

### Surface plasmon resonance (SPR) analysis

SPR measurements were performed on a BIAcore X100 (Cytiva). The recombinant human RANK/Fc Chimera or OPG was immobilized by standard amine-coupling chemistry on a research-grade CM5 sensor chip (Cytiva). RANK/Fc and OPG were resuspended at 50 µg/ml and 10 µg/ml, respectively, in 10 mM sodium acetate pH 4.8, and injected for 420 seconds at a flow rate of 10 µl/min on the first cell of the activated sensor chip. Then, the surface was deactivated [53]. In these conditions, the immobilization of 3000–5000 resonance units (RU) was routinely obtained for the two proteins. On the second cell, used as a negative control for blank subtraction, the activation/deactivation procedure was performed without proteins. The effective immobilization of the two proteins and their binding availability was validated by successful binding assays with RANKL (not shown). For binding analysis, increasing concentrations of E123CaG were resuspended in 10 mM HEPES, 150 mM NaCl, 3 mM EDTA, 0.05% surfactant P20 (pH 7.4) and injected onto the RANK- or OPG-containing surfaces for 180 seconds at a flow rate of 30 µl/min to allow their association and then washed until dissociation was observed.

### Western blot

#### Cell-conditioned media

Serum-free media conditioned by sub-confluent cells (48 h incubation) were collected, centrifuged at 3000 rpm for 15 min at 4 °C, frozen, and stored at −20 °C until analysis.

#### Cell lysates

Cells were lysed with RIPA buffer (Thermo Fisher) containing Roche cOmplete protease inhibitor cocktail and phosphatase inhibitor phosSTOP (Sigma-Aldrich). The collected lysates were maintained on ice for 1 h and then centrifuged at 10,000×*g*, 10 min at 4 °C, and the supernatant was frozen and stored at −20 °C until analysis.

After normalization for protein content, proteins were separated by gel electrophoresis (Mini-PROTEAN TGX gels 4–15%, Bio-Rad, Segrate, Italy) under reducing conditions and transferred to nitrocellulose membranes by Trans-Blot Turbo System (Bio-Rad). After blocking with 5% ECL PrimeTM blocking reagent (Cytiva) in Tris-buffered saline (TBS) 0.1% Tween-20, membranes were incubated overnight at 4 °C with primary antibodies in TBS 0.1% Tween-20 2% ECL PrimeTM blocking reagent. The primary antibodies used recognized TSP-1 (clone A6.1, MA5-13398, Invitrogen, 1:300), GFP (NB600-308, Novus Biologicals, 1:1000), phospho-SAPK/JNK and SAPK/JNK (9251 and 9252, Cell Signaling Technology, 1:1000), phospho-p38 MAPK and p38 MAPK (9216 and 8690, Cell Signaling Technology, 1:1000), and tubulin (T9026, Sigma-Aldrich, 1:1000). After incubation with peroxidase-labeled secondary antibodies followed by ECL Western blotting substrate (Cytiva), signals were detected using Odyssey FC Imaging System (LiCor, Lincoln, NV), and bands quantified using ImageJ software. Original western blots are presented as Supplemental Material.

### Cells and transfection

The mouse triple negative mammary carcinoma 4T1.2 cells were maintained in RPMI 1640 medium (Euroclone, Milan, Italy) supplemented with 10% FBS (Gibco, Thermo Fisher) and 2 mM L-glutamine (Euroclone).

4T1.2 cells were transfected to express and secrete the E123CaG fragment (ECaG cells). The cDNA coding for E123CaG, attached to an N-terminus IgK-leader sequence for secretion (Genscript, Leiden, the Netherlands) was inserted into pcDNA3.1 + C-eGFP vector for C-terminally GFP-tagging. The empty vector was used to transfect control cells (EV cells). Cells were transfected using Lipofectamine (Invitrogen), followed by selection with 500 µg/ml geneticin (G418, Invitrogen, Thermo Fisher) and enrichment for GFP positivity by FACS. Expression and secretion of the fragment by 4T1.2-ECaG cells were confirmed in conditioned media and cell lysates by western blot analysis, using an anti-GFP antibody (NB600-308 Novus Biologicals, Bio-Techne, Milano, Italy 1:1000).

The murine macrophage-like RAW 264.7 cell line (ATCC TIB-71TM) was maintained in DMEM 10% FBS and 2 mM L-glutamine and detached by gentle cell scraping.

Stocks of cells were stored frozen in the vapor phase of liquid nitrogen, and cells were used within 1 month of thawing. Cells were routinely tested and found free of mycoplasma infection.

### Osteoclastogenesis assay

#### BM precursors

Bone marrow (BM) cells were collected from the femurs and tibiae of six to eight-week-old female BALB/c mice by repeatedly flushing the marrow cavity with MEM-Alpha. After filtration with a cell strainer (40 μm, Falcon, Fisher Scientific) to remove debris, cells were collected by centrifugation (400× *g*, 5 min, 4 °C), resuspended in Red Blood Cell Lysing Buffer (Hybri-Max, Sigma-Aldrich), and incubated for 5 min on ice. The reaction was stopped by FBS. Cells were then centrifuged, resuspended in complete medium (MEM-Alpha supplemented with 10% FBS, GlutaMAX, ribonucleosides, deoxyribonucleosides, and 1% Penicillin-Streptomycin), plated in 100 mm tissue culture dishes, and incubated at 37 °C overnight. Non-adherent cells were then collected and plated in complete MEM-Alpha into 96-well plates (200,000 cells/well), in the presence of 50 ng/ml macrophage-colony stimulating factor (M-CSF, R&D System) and 100 ng/ml RANK-L with or without the indicated doses of TSP-1, fragments, or OPG. Media and factors were changed after 2 days. At day 5, cells were fixed by 4% paraformaldehyde and stained for TRAP (Leukocyte Acid Phosphatase TRAP Kit, Sigma-Aldrich). Osteoclasts, TRAP-positive cells with more than three nuclei, were counted in a blinded manner.

#### RAW 264.7 cells

Cells were plated in complete MEM-Alpha medium in 96-well plates (7500 cells/well) supplemented with 50–100 ng/ml RANK-L and the indicated concentration of TSP-1, fragments, or OPG. Media and factors were changed after 2 days. At day 4, large multinucleated osteoclasts were visible and counted as described above.

### RANK signaling analysis

RAW264.7 cells were seeded into 24-well plates (45000 cells/well) in complete MEM-Alpha 10% FBS. After 72 h, cells were incubated with E123CaG (1 µM) in MEM-Alpha w/o FBS for 3 h, then exposed to RANK-L (50 ng/ml) in the same medium for 20 min (early signaling, p38 and JNK) or 48 h (late signaling, NFATc1). At the end of incubation, cells were washed twice in PBS, lysed, and proteins analyzed by western blotting, as above.

### Immunofluorescence

#### Actin

To detect the formation of the acting ring, BM-derived precursors were plated (600000 cells/well) in 18-well ibiTreat micro-slides (Ibidi, Giemme, Milan, Italy), in complete MEM-Alpha and induced to differentiate by M-CSF and RANKL as described above, in the presence or not of E123CaG (0.5 µM). When multinucleated osteoclasts appeared (day 5), cells were fixed with 2% PFA in PBS 4% sucrose for 10 min at room temperature, permeabilized with 0.1% Triton X-100 for 3 min, and then incubated for 30 min with PBS 3% BSA at room temperature. Cells were then stained with Alexa Fluor 488 Phalloidin for 1 h at room temperature, and nuclei were counterstained with DAPI for 5 min. Images (63X) were taken using a Leica SP8 confocal microscope (Leica Microsystems, Wetzlar, Germany).

#### NFATc1

RAW 264.7 cells were grown in 18-well ibiTreat micro-slides (Ibidi). E123CaG (1 μM) and RANK-L (50 ng/ml) in medium with 5% FCS were added and incubated for 48 h. Cells were then fixed with 2% PFA in PBS 4% sucrose for 10 min at room temperature, permeabilized with 0.1% Triton X-100 for 3 min, and then incubated for 30 min with PBS 3% BSA at room temperature. Cells were then stained with the anti-NFATc1 primary antibody (Sc-7294, Santa Cruz Biotechnology, Heidelberg, Germany 1:200) in PBS 1% BSA overnight at 4 °C, followed by donkey anti-mouse Cy3 secondary antibody for 1 h at room temperature. Nuclei were counterstained with DAPI. Images (40X) were taken using a Leica SP8 confocal microscope. NFATc1 signal intensity in the nucleus and cytoplasm was then quantified with ImageJ and expressed as a nuclear to cytoplasmic ratio.

### Proteolytic degradation of TSP-1

RAW 264.7 cells were induced to differentiate as above. TSP-1 (0.5 μg/well) in MEM-Alpha without FCS was added and incubated for 24 h at 37 °C. The serine protease inhibitors AEBSF and leupeptin (Sigma-Aldrich), the MMP inhibitor batimastat (MCE, DBA Milan, Italy), antibodies against HTRA1 (MAB2916, R&D System, 20 µg/ml) or control IgG (Invitrogen, 20 µg/ml) were added as indicated. The conditioned media were collected, centrifuged at 3000 rpm for 15 min at 4 °C, and subjected to western blot analysis using the monoclonal anti-TSP-1 antibody, clone A6.1.

### Proteolytic degradation of OPG

Biotin-labeled OPG (0.08 μg/well) in MEM-Alpha without FCS was added to differentiated or non-differentiated RAW 264.7, in the absence or presence of E123CaG (1 μM), and incubated for 24 h at 37 °C. The supernatant was then collected, centrifuged at 3000 rpm for 15 min at 4 °C. In other experiments, biotin-labeled OPG (0.1 μg) was incubated with recombinant HTRA1 (R&D System, 0.1 µg) for 24 h at 37 °C. Degradation of OPG was evaluated by western blot analysis with ExtrAvidin-Peroxidase.

### In vivo procedures

Procedures involving animals and their care were conducted in conformity with the institutional guidelines, that comply with national (Lgs 26/2014) and European Union directives laws and policies (EEC Council Directive 2010/63), in line with guidelines for the welfare and use of animals in cancer research. Studies were approved by the Mario Negri Institute Animal Care and Use Committee and by the Italian Ministry of Health (authorization no. 125/2016-PR). Six- to eight-week-old female BALB/c (Charles River, Lecco, Italy), maintained under specific-pathogen-free conditions and handled using aseptic procedures, were used.

#### Bone metastasis model

Parental, ECaG, and EV cells (100,000 cells/100 μl in HBSS) were injected into the caudal artery (CA) of anesthetized mice, applying a pressure sufficient to counteract blood pressure. Mice were monitored three times a week, received analgesics when symptoms of bone metastasis (difficulty in locomotion) appeared, and were euthanized at the first signs of suffering.

#### Micro-CT analysis of bones

Micro-CT scans before (T0) and 7–14–21 days after tumor cell injection were acquired using a high-performance micro-CT (SkyScan 1076 micro-CT, Bruker, Billerica, MA), equipped with a 4000 × 2300-pixel X-ray detector. Scans were conducted at maximum resolution (9 μm isotropic voxel size). Acquisition settings were: X-ray source voltage 50 kV, source current 200 μA, and 0.5 mm aluminum filter. The exposure time per projection was 1400 ms, with rotational steps of 0.8°, resulting in a total of 250 projections over a 15-min scan duration. Volume reconstruction was carried out using a filtered back-projection algorithm (NRecon software, version 1.7.4.6, Bruker). After tridimensional reconstruction of the right femur and tibia, the structural parameters were evaluated by BoneJ (ImageJ plugin) in a blinded manner [54]. To achieve the correct orientation of the reconstructed bones, the “Moments of inertia” from BoneJ was applied to each image, and then the quantitative analysis of bones was performed, selecting specific ROIs covering the regions of interest. The following parameters were considered: (i) bone volume fraction BV/TV (bone matrix volume/total bone volume); (ii) bone surface, evaluated by “Marching cubes” algorithm, which calculates the area of a specific surface by constructing a triangular surface mesh [55]; (iii) Tb.Th. (trabecular thickness), average thickness of trabecular bone voxels, defined by the diameter of the largest sphere which encloses that point and is entirely bounded within the bone); (iv) Tb.Sp. (trabecular separation or space), space between trabeculae. For each parameter, the results are expressed as the difference (delta) between the value at day 14 and day 0 after the injection of tumor cells.

### Statistical analysis

For in vivo studies, at least 6 mice were enrolled in each experimental group, as determined based on prior studies and power calculations, as the sample size necessary to detect an effect size of 1.7 with 80% power and 5% significance level. Before injection, mice were randomized by body weight. Two mice were excluded from the analysis due to technical issues. Non-parametric Mann–Whitney test was used to compare two groups, and one-way ANOVA, followed by Dunnett’s or Tukey’s multiple comparison test for three or more groups. Welch’s ANOVA was used when the assumption of equal variances (Barlett’s test) was violated. For grouped analysis, with two independent variables, two-way ANOVA followed by Tukey’s test was used. A *P* values less than 0.05 was considered statistically significant. Statistical analysis was carried out using GraphPad Prism version 10.0.3 software (GraphPad, La Jolla, CA).

## Supplementary information

Supplementary Material

Full uncropped western blots

Reproducibility checklist

## Data Availability

The data generated in this study are available within the article and its supplementary data files. Data and materials will be made available upon request and, if applicable, material transfer agreements.
