# Supplementary material for: TSP-1 interaction with RANK and OPG: implications for bone remodeling and osteolytic bone metastasis
Source: Cell Death Dis. 2026 Mar 21;17(1):332. doi: 10.1038/s41419-026-08600-9 (PMC13039915; doi:10.1038/s41419-026-08600-9)
Supplement: Supplementary file 1 — Supplementary Material [file 41419_2026_8600_MOESM1_ESM.docx]

**TSP-1 interaction with RANK and OPG: implications for bone remodeling and osteolytic bone metastasis**

Laura Carminati et al.

**Supplementary material**

**_________________________________________________________**

** A B**

**
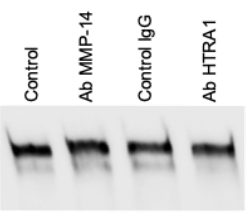
**
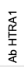


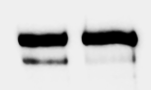


**Supplementary Figure 1. Role of HTRA1 in osteoclast-mediated proteolytic processing of TSP-1**

A) Cleavage of TSP-1 by recombinant HTRA1. TSP-1 (0.5 μg) was incubated with HTRA1 (0.25 μg) for 24 hours at 37°C.

B) Proteolytic cleavage of TSP-1 by osteoclasts is prevented by antibodies against HTRA1 but not by antibodies against the metalloprotease MMP-14 (MAB3328 Sigma-Aldrich, 20 µg/ml). Part of this image in shown in Fig 1I of the main manuscript).

The formation of fragments was analyzed by Western blot analysis with A6.1 antibody.

**B**

**A**

Bound RANKL (%)

E123CaG (1)

E123CaG (0.3)

Control

Bound RANKL (%)

E123CaG (1)

Control

**Supplementary Figure 2. E123CaG does not prevent the biding of RANKL to RANK**

**A)** Effect of the indicated concentration of E123CaG (in µM) on RANKL/RANK binding assessed with the RayBio Assay Kit (RayBiotech Life, Peachtree Corners, GA), performed according to the manufacturer’s instructions. **B**) Effect of E123CaG of the binding of Eu-labeled RANKL to RAW 264.7 cells. RANKL was labeled with europium using the DELFIA Eu-N1 ITC chelate labeling Kit (Perkin Elmer), followed by isolation of labeled RANKL (Eu-RANKL) by ZebaTM Spin Desalting Columns. Eu-RANKL (1 ng/well) was added to confluent RAW264.7 cells in 96-well plates in DMEM supplemented with 0.15 % gelatin and 25 mM HEPES in the presence or not of E123CaG (1 μM). After a 2 hours-incubation at 4°C, wells were washed three times with DMEM-gel and once with PBS, incubated with DELFIA Enhancement Solution, and bound Eu-RANKL was quantified measuring time-resolved fluorescence. Data are the percentage of control binding (mean ± SD of values from one experiment representative of at least two).

***
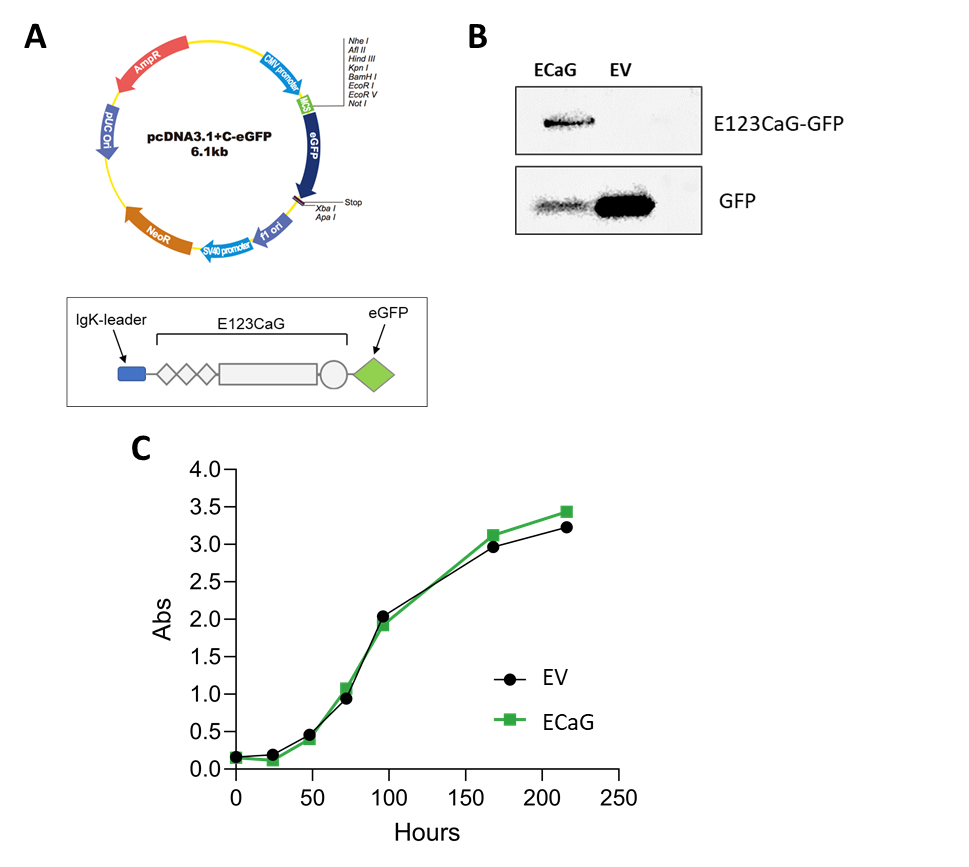
***

**Supplementary Figure 3. The ECaG cell model**

4T1.2 mouse breast cancer cells were engineered to express and secrete the TSP-1 fragment E123CaG. **A**) schematic representation of the *pcDNA3.1+C-eGF* vector and the E123CaG sequence linked N-terminally to an IgK-leader sequence for secretion and C-terminally to GFP. Control cells were transfected with the empty vector. **B)** WB analysis using anti-GFP antibody showing the presence of GFP-tagged E123CaG protein in the conditioned media of 4T1.2 cells transfected to express E123CaG (ECaG) but not in control cells (EV), that express only GFP. **C**) In vitro proliferation of 4T1.2 cells transfected with E123CaG or EV. Cells were seeded in 96-well plates (2000 cells/well) in complete medium. At the indicated time points, cells were stained with Crystal Violet staining solution and absorbance was measured at 595 nm (one experiment representative of three).

**Supplementary Figure 4. Orthotopic growth of 4T1.2 E123CaG cells**

4T1.2 cells expressing (ECaG) or not (EV) E123CaG (10^5^ cells/10 μl) in Hank’s Balanced Salt solution were injected in the mammary fat pad (mfp) of BALB/c mice. Primary tumors were measured with a caliper, and tumor volume (mm^3^) was calculated as (length × width^2^)/2 at the indicated time point (mean ± SEM, n=9).


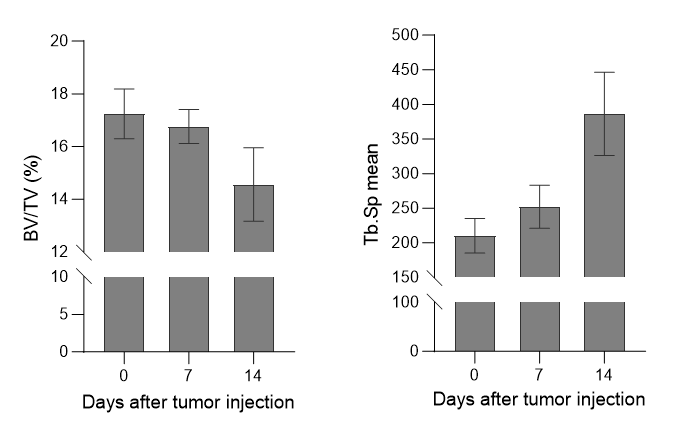


**Supplementary Figure 5. Bone degradation associated with bone metastasis**

4T1.2 cells (10^5^) were injected into the caudal artery of mice. To monitor the formation of osteolytic metastasis, in vivo micro-CT scans of hind limbs were performed before injection (time 0) and after 1 and 2 weeks. The scans were analyzed as described in Materials and Methods. Bone degradation is expressed as loss of bone matrix (BV/TV% = bone volume/total volume ratio) and increased space among trabeculae (Tb.Sp. mean, in µm).
