# Supplementary figures and images for: TSP-1 interaction with RANK and OPG: implications for bone remodeling and osteolytic bone metastasis
Source: Cell Death Dis. 2026 Mar 21;17(1):332. doi: 10.1038/s41419-026-08600-9 (PMC13039915; doi:10.1038/s41419-026-08600-9)

ORIGINAL WESTERN BLOT

FIGURE 1 G, H, I

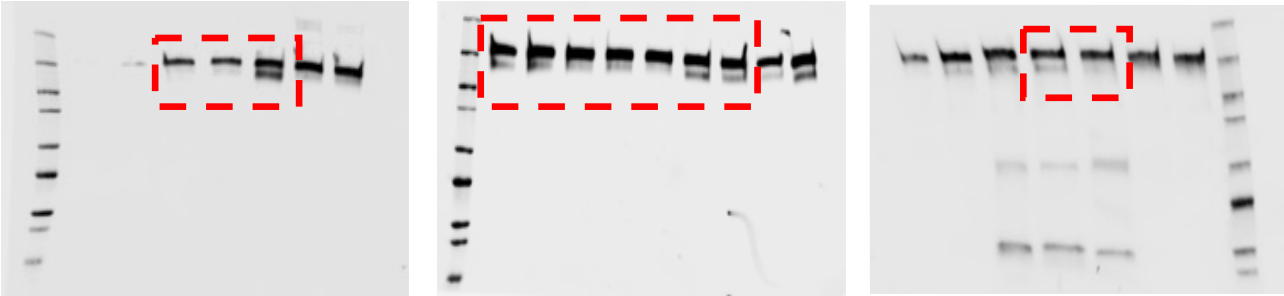

FIGURE 3 A

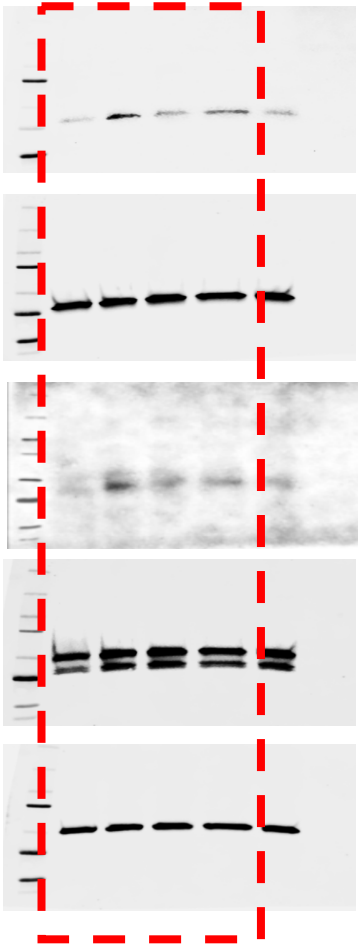

FIGURE 5 C, D

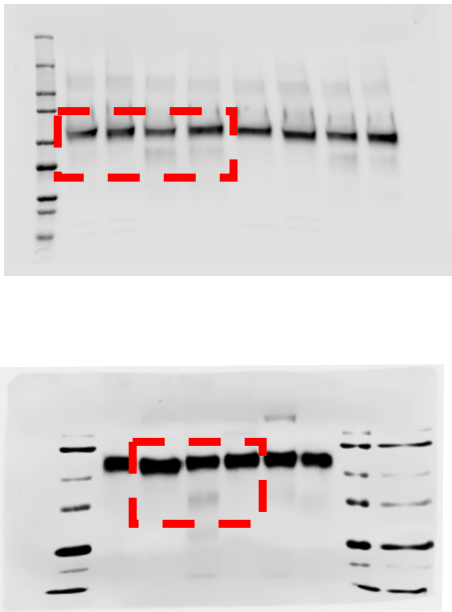

Supplement: Supplementary file 2 — Full uncropped western blots [file 41419_2026_8600_MOESM2_ESM.pdf]
